# Supplementary material for: Exercise-Induced Arrhythmia or Munchausen Syndrome in a Marathon Runner?
Source: Diagnostics (Basel). 2023 Sep 12;13(18):2917. doi: 10.3390/diagnostics13182917 (PMC11340689; doi:10.3390/diagnostics13182917)
Supplement: Supplementary file 1 [file diagnostics-13-02917-s001.zip › diagnostics-2513665-SM.pdf]

Table of Contents

S1. ECG test result 1

S2. Holter ECG results... 1

S3. Echocardiography results... 2

S4. MRI results 3

S5. Cardiac electrophysiology study.. 8

Table S1. The athlete’s personal records. 10

S1. ECG test result

Figure S1. ECG test result

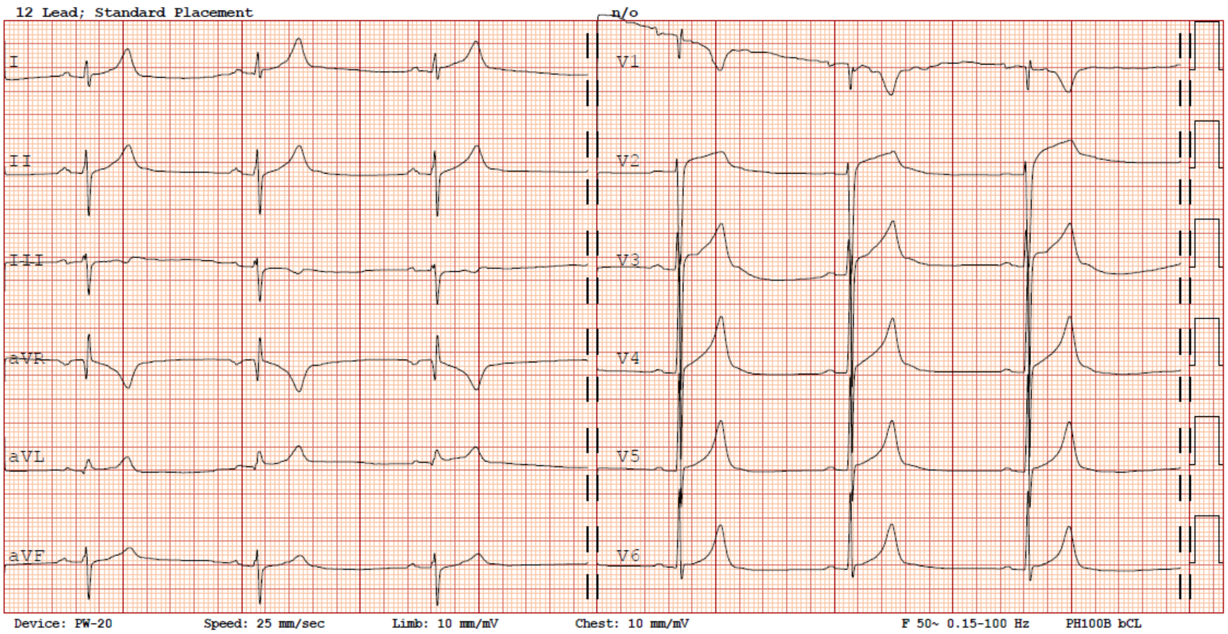

Sinus bradycardia 40/min. Left axis deviation. Left anterior hemiblock.

Repolarization abnormalities: early repolarization, ST elevations, high T waves

S2. Holter ECG results

A series of Holter ECGs were performed in 2022-2023, also during competitions. Sinus rhythm averaging 53-55 bpm (31 min-176 max bpm) was observed, with no significant heart rhythm abnormalities. Single ventricular and supraventricular beats were noted. No conduction disturbances, pathological pauses, or specific ST changes were observed. The nocturnal bradycardia min. 31 beats/min. Periodically (especially during high physical activity) there were recordings with a lot of artifacts, preventing correct interpretation.

S3. Echocardiography results

Video S3. Echocardiography results

A. Parasternal long-axis view

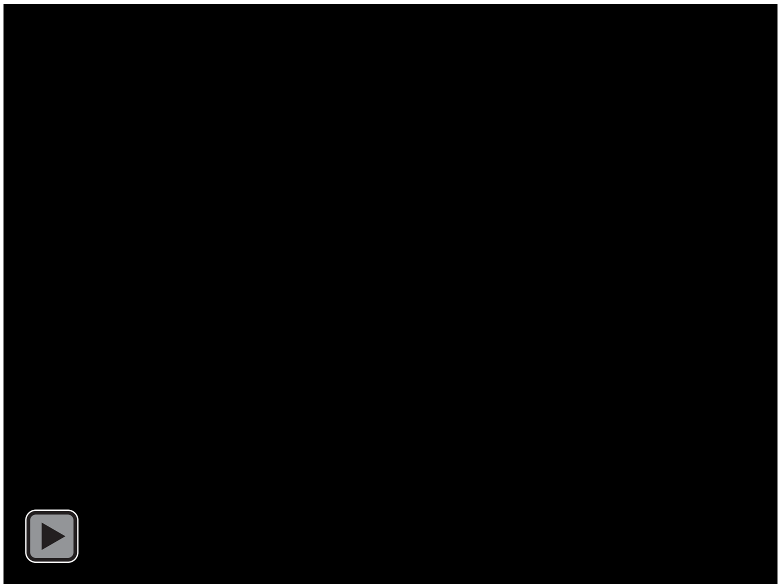

B. Apical 3-Chamber View (Long-Axis View)

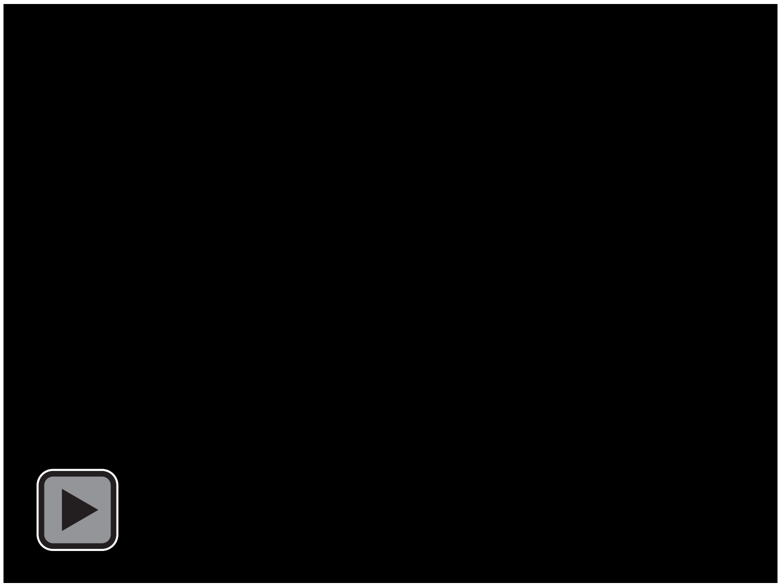

The left ventricle diastolic diameter is 48 mm. The left wall thickness increased to 13-15 mm, especially in the septal region. There is normal muscle contractility. LVEF 65%, E/A 1.9, E/E' 4.4. The right ventricle is not enlarged and exhibits normal contractility. TAPSE 2.9 cm, W' 11cm/sec. The ascending aorta goes from 39 mm to 33 mm in the aortic arch. The valves do not show any significant morphological changes. There is slight mitral regurgitation and minor tricuspid regurgitation. The right ventricular systolic pressure is 25 mmHg. LA area 27 cm^2, RA area 27 cm^2.

Abbreviations:

LA – left atrium, LVEF – left ventricle ejection fraction, RA – right atrium

S4. MRI results

Figure S4 MRI. results

A. MRI – Stress perfusion CMR without perfusion defects

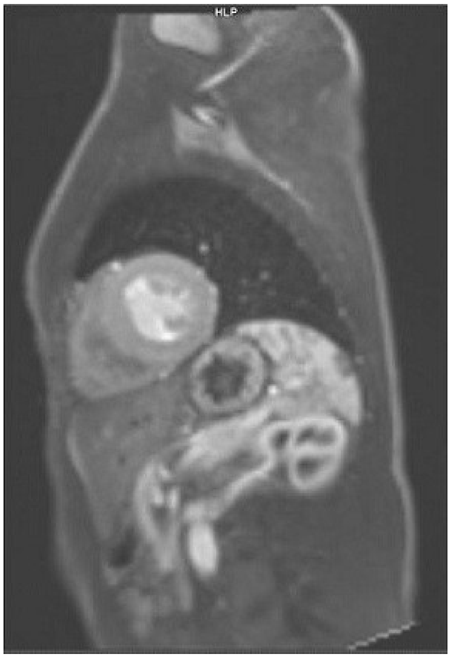

B. MRI – short-axis view without late gadolinium enhancement

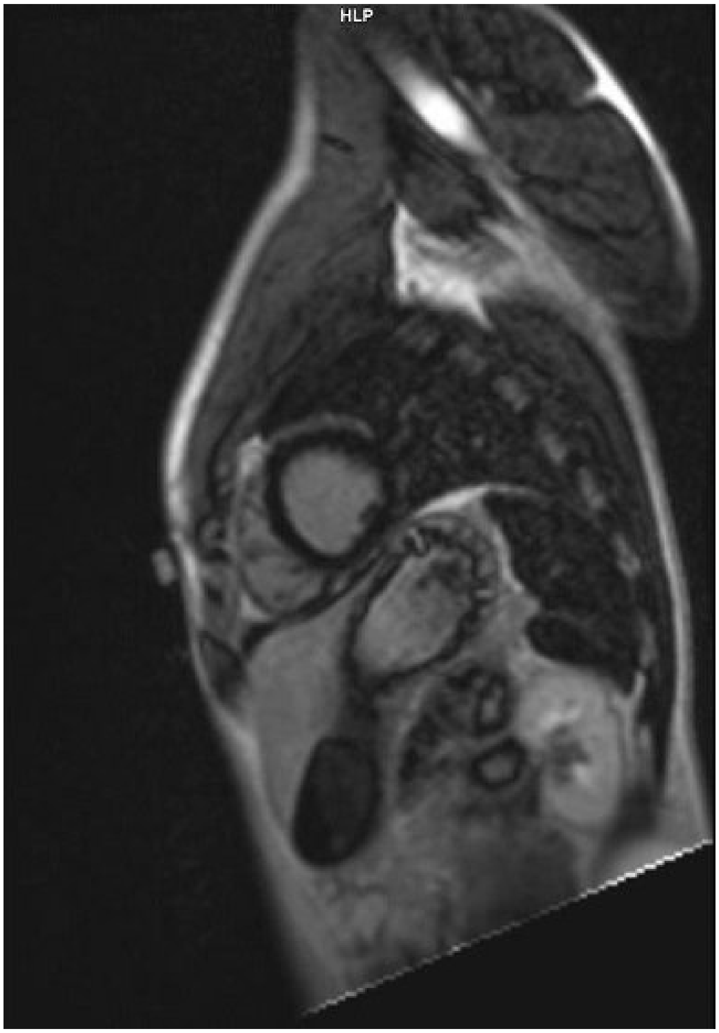

C. MRI – Perfusion normal

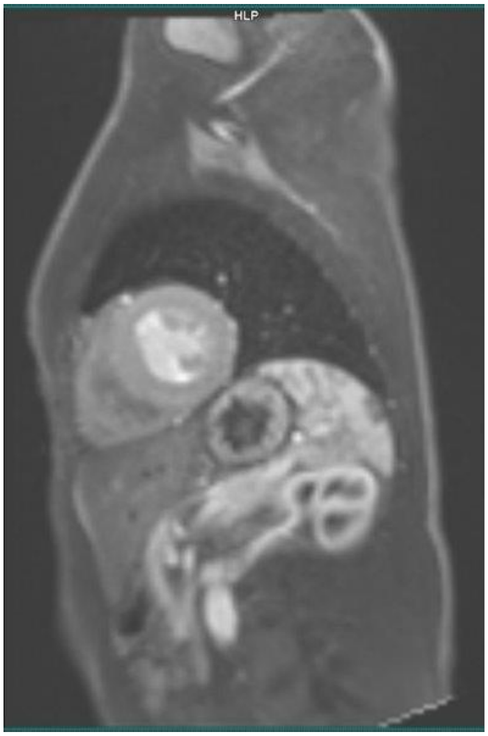

D. MRI – normal 4-chamber view without late gadolinium enhancement

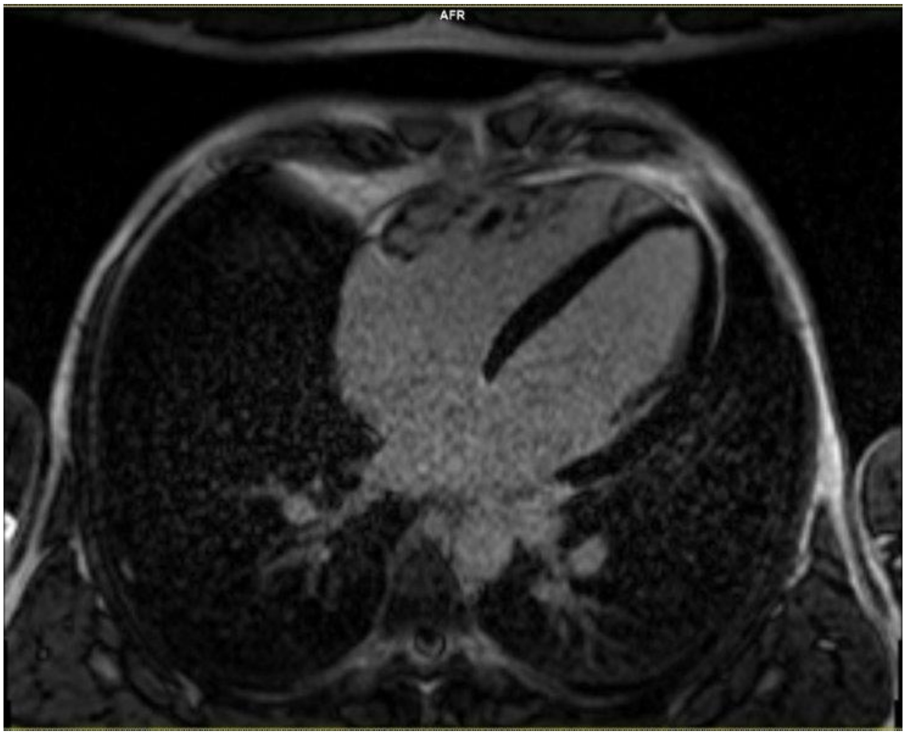

Video S4. MRI results

A. 4-chamber cine images demonstrating no motion abnormality

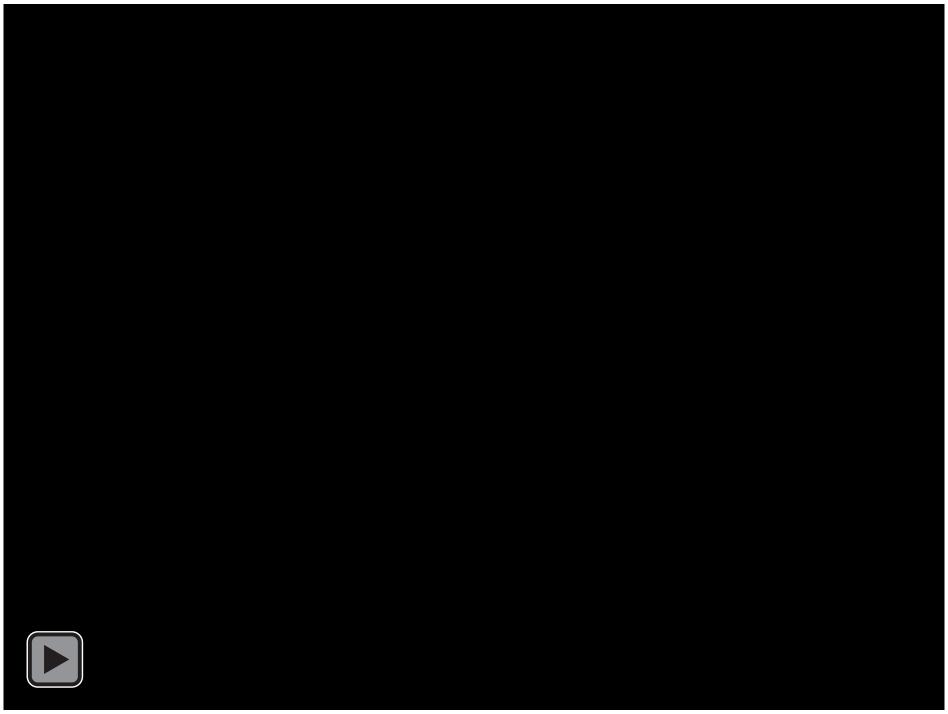

B. MRI – short-axis cine images

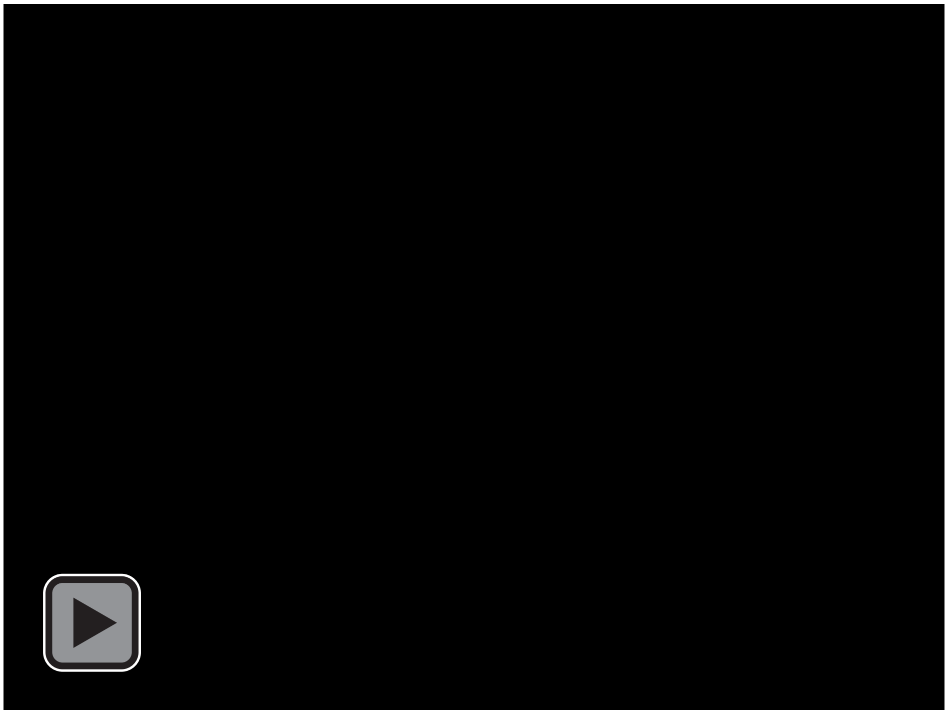

Left ventricle (LV)—the LV has an increased indexed volume. The contractility of the LV muscle (EF=63%) is correct. There are no regional contractility disorders. The basal antero-septal segment is thickened (up to 14 mm, may be partly overestimated due to difficulties in distinguishing the interventricular septum and the marginal band). The remaining left ventricular muscle thickness is assessed as normal. The left ventricular muscle mass increased (mainly due to its enlargement).

Right ventricle (RV)—the RV has an increased indexed volume. There is normal muscle contractility (EF=61%) and no regional contractility disorders. The right ventricular muscle is of normal thickness.

Perfusion assessment (regadenoson administration) —there are no myocardial perfusion losses.

Late post-contrast enhancement (LGE) —thrombi were not visualized in the early phase after administration of the gadolinium contrast agent. There were no areas of LGE.

Both atria are enlarged (LA 29 cm2, RA 37 cm^2).

The heart valves exhibit normal morphology and function, with slight mitral and tricuspid regurgitation.

The aortic root is 43 mm, with normal width of the ascending aorta and pulmonary artery (30 mm).

Pericardium—the pericardium is of normal thickness, and there is a physiological amount of fluid in the pericardial cavity.

Conclusions:

1. The heart cavities are symmetrically enlarged (feature of an athlete's heart).

2. The muscle contraction of both ventricles is normal.

3. The basal antero-septal segment of the LV is thickened (up to 14 mm but can be affected by the presence of the marginal band).

4. There are no areas of left ventricular ischemia during hyperemia.

5. There are no areas of left ventricular fibrosis/necrosis.

6. The aortic root is widened (up to 43 mm).

S5. Cardiac electrophysiology study

Figure S5. Cardiac electrophysiology study

A: Invasive electrophysiologic study (EPS) in the patient after episodes of fainting during maximum competitive effort preceded by self-reported accelerated heartbeat/palpitations events. Programmed electrical stimulation of the right ventricular apex after intravenous administration of a beat-agonist

S1-S1-S2-S3-S4 400 ms-200 ms -200 ms -200 ms; S2 pacing falls in RV refractory period.

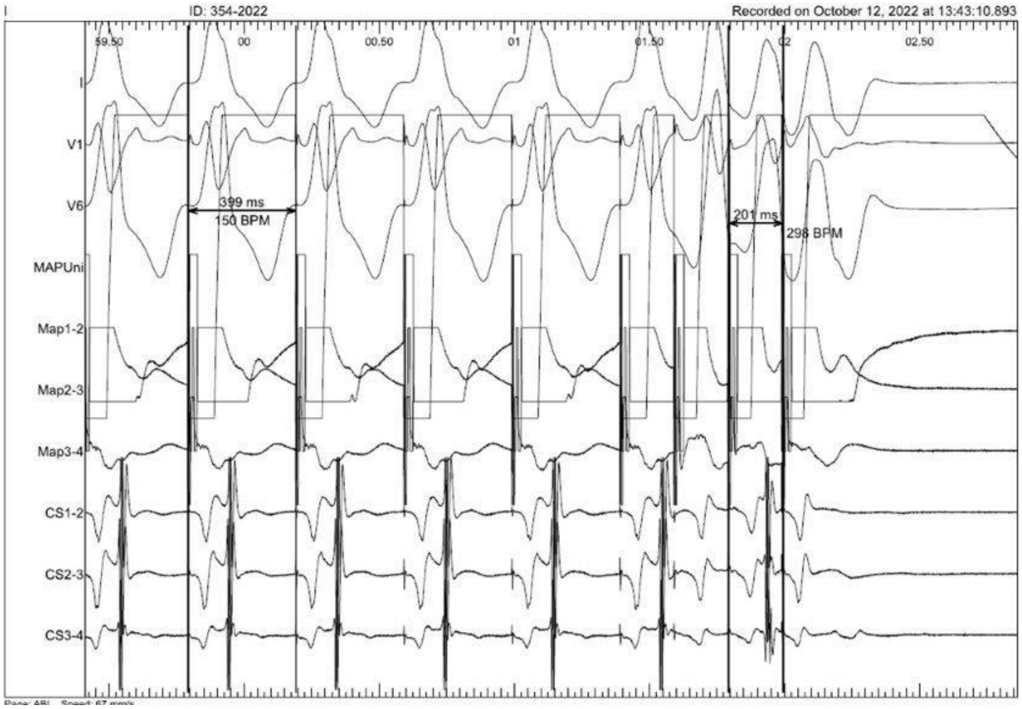

B: Invasive electrophysiologic study (EPS) in the patient after episodes of fainting during maximum competitive effort preceded by self-reported accelerated heartbeat/palpitations events

Programmed electrical stimulation of the right ventricular apex after intravenous administration of a beat-agonist

S1-S1-S2-S3-S4 400 ms-190 ms - 190 ms - 190 ms; S2 and S4 pacing stimuli fall in RV refractory period, S1-S2 coupling interval 380 ms gives evoked ventricular contraction.

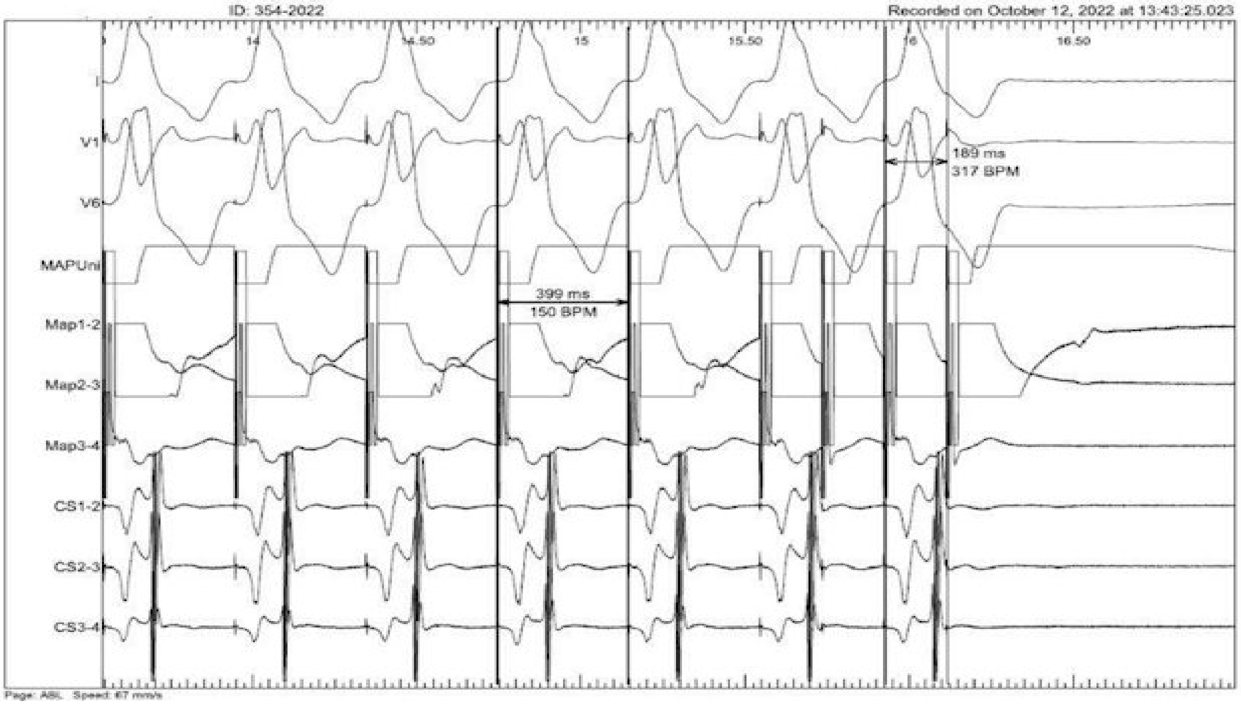

Table S1. The athlete's personal records.

Marathon	2:17:22	Limassol, Cyprus 2014
Half Marathon	1:06:14	Warsaw, Poland 2014
15 km Street	47:43	Warsaw, Poland 2020
10 km Street	30:07	Radom, Poland 2014
5 km Street	14:23	Grand Junction, USA 2013
50 km Stadium	2:55:47	Katowice, Poland 2022 (Polish record)
10 km Stadium	30:03:78	Międzyzdroje, Poland 2005
10 km cross-country	31:09	Fayetteville, AR, USA 2010
8 km cross-country	24:49	Louisville, KY, USA 2010
5 km Stadium	14:30:40	Białystok, Poland 2004
3 km Obstacles	9:05:91	Louisville, KY, USA 2011
3 km Stadium	8:21:72	Warsaw, Poland 2006
1 mile Stadium/Hall	4:14:15	Indianapolis, IN, USA 2011
1 mile Street	4:18	Little Rock, AR, USA 2013
1500 m Stadium	3:54:75	Poznań, Poland 2004
1000 m Stadium	2:27:05	Olsztyn, Poland 2005
800 m Stadium	1:56:30	Olsztyn, Poland 2005
